# Right ventricular stress-induced perfusion defects and late gadolinium enhancement in coronary artery disease

**DOI:** 10.1186/1532-429X-16-S1-P210

**Published:** 2014-01-16

**Authors:** Michael W Milks, Bharathi Upadhya, Gregory Hundley, R Brandon Stacey

**Affiliations:** 1 Internal Medicine - Cardiology, Wake Forest Baptist Health, Winston Salem, North Carolina, USA

## Background

Due to relatively thin walls, assessment of right ventricular (RV) perfusion defects has remained challenging during vasodilator stress perfusion with cardiovascular magnetic resonance (CMR). Previously, RV perfusion defects observed during radionuclide vasodilator stress have been found associated with adverse cardiovascular (CV) prognosis. However, to date, few data are available regarding the interpretation of RV perfusion defects during vasodilator stress and late gadolinium enhanced CMR.

## Methods

Among 61 individuals aged 59 ± 11.8 years, (10% African-American, 47% women, 81% hypertensive, 39% diabetic, and 76% hyperlipidemic) who underwent cardiac MRI adenosine stress testing prior to cardiac catheterization, we assessed coronary artery stenoses, mortality (assessed by chart review and the social security death index) and the presence of both stress and rest perfusion defects as well as the presence of late gadolinium enhancement by personnel blinded to each aspect of the study. Adjusting for age, race, and gender, a logistic regression model was used to describe the potential association between significant (70% or greater) coronary stenoses and RV perfusion defects. Adjusting for the same covariates, a Cox proportional hazard model was used to describe the potential association between mortality and the presence of RV late gadolinium enhancement (RVLGE).

## Results

Of the 61 cases, 26% (n = 16) had no obstructive disease, 30% (n = 18) had single vessel disease, 21% (n = 13) had double vessel disease, 23% (n = 13) had multi-vessel disease, 36% (n = 22) had proximal LAD disease, 39% (n = 24) had proximal circumflex disease, and 43% (n = 26) had proximal RCA disease. Right ventricular stress-induced perfusion defects were positively associated with proximal RCA and LAD stenoses (p < 0.01). There was a trend toward a significant positive association (p = 0.10) with distal RCA disease, but LCx artery stenosis was associated with not having a stress-induced perfusion defect within the RV myocardium (p = 0.024). The presence of RVLGE was associated with mortality (p = 0.009), but 77% of those with RVLGE also had LV late gadolinium enhancement.

## Conclusions

Proximal RCA and LAD stenoses are positively associated, whereas LCx stenoses are negatively associated, with right ventricular stress-induced perfusion defects identified by cardiac MRI stress testing. Right ventricular late gadolinium enhancement was associated with increased mortality, but further studies will be needed to determine if this is independent of left ventricular late gadolinium enhancement.

## Funding

Only departmental funds were needed.

**Figure 1:**
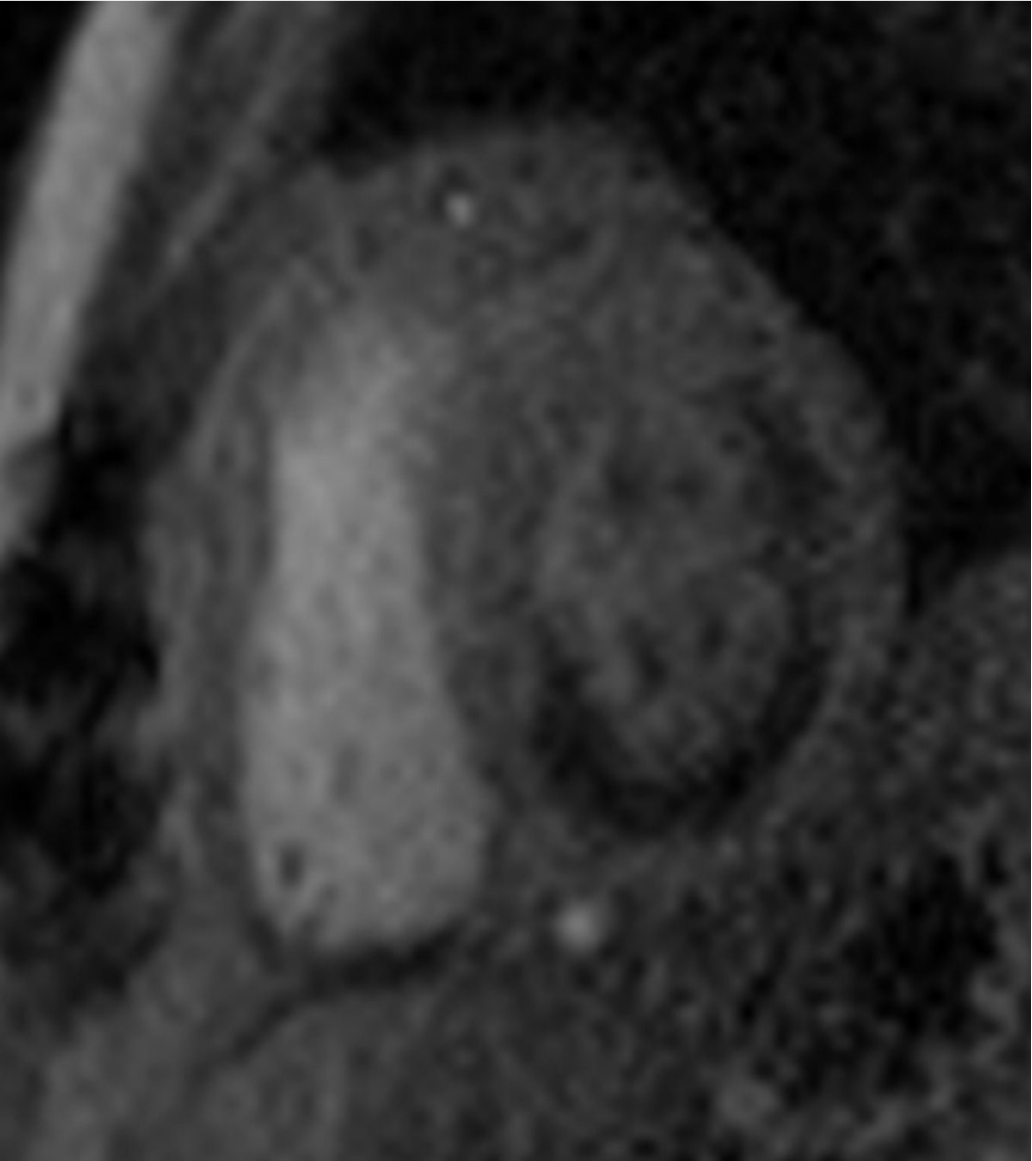
**Short axis slice of right ventricular stress-induced perfusion defect**.

**Figure 2:**
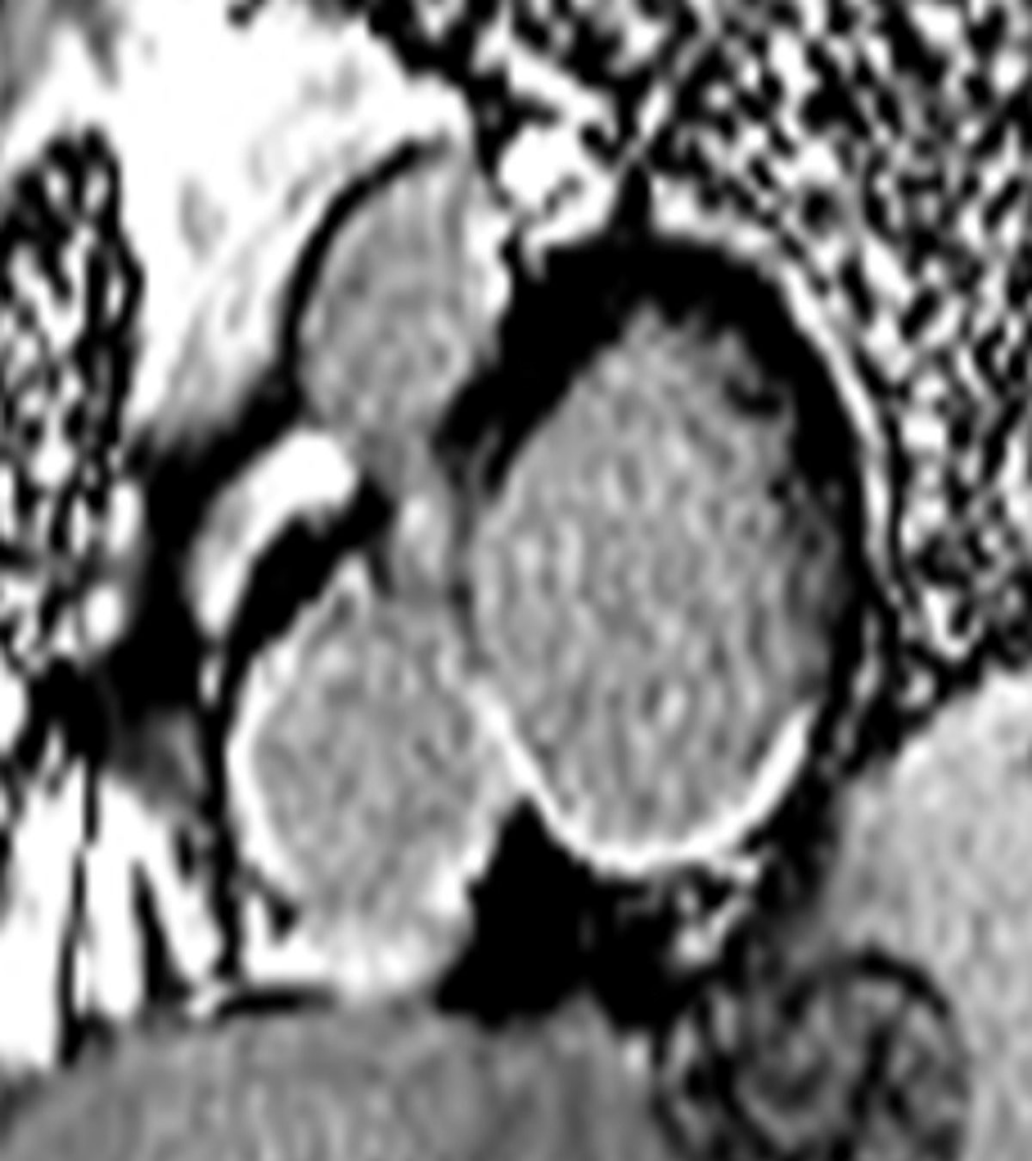
**Short axis slice of right ventricular late gadolinium enhancement**.

